# Effectiveness of acoustic lures for increasing tropical forest understory bat captures

**DOI:** 10.1002/ece3.8775

**Published:** 2022-03-31

**Authors:** Oliver Aylen, Philip J. Bishop, Rodzay bin Haji Abd Wahab, T. Ulmar Grafe

**Affiliations:** ^1^ 2495 Department of Zoology University of Otago Dunedin New Zealand; ^2^ 7800 School of Biological, Earth and Environmental Sciences (BEES) University of New South Wales Sydney Australia; ^3^ 37597 Institute for Biodiversity and Environmental Research Universiti Brunei Darussalam Gadong Brunei Darussalam; ^4^ 37597 Faculty of Science Universiti Brunei Darussalam Gadong Brunei Darussalam

**Keywords:** acoustic lure, bats, bioacoustics, Borneo, capture techniques, Chiroptera, echolocation, monitoring techniques

## Abstract

Bats are the most diverse mammalian order second to rodents, with 1400+ species globally. In the tropics, it is possible to find more than 60 bat species at a single site. However, monitoring bats is challenging due to their small size, ability to fly, cryptic nature, and nocturnal activity. Recently, bioacoustic techniques have been incorporated into survey methods, either through passive acoustic monitoring or acoustic bat lures. Lures have been developed on the premise that broadcasting acoustic stimuli increases the number of captures in harp traps or mist nets. However, this is a relatively new, niche method. This study tested the efficacy of two commonly used acoustic bat lure devices, broadcasting two different acoustic stimuli, to increase forest understory bat captures in the tropics. This is the first time an acoustic bat lure has been systematically tested in a tropical rainforest, and the first study to compare two lure devices (Sussex AutoBat and Apodemus BatLure). Using a paired experimental design, two synthesized acoustic stimuli were broadcasted, a feeding call and a social call, to understand the importance of the call type used on capture rates and genus‐specific responses. Using an acoustic lure significantly increased capture rates, while the type of device did not impact capture rates. The two acoustic stimuli had an almost even distribution of captures, suggesting that the type of call may be less important than previously thought. Results indicate a possible deterrent effect on *Rhinolophous* sp., while being particularly effective for attracting bats in the genera *Murina* and *Kerivoula*. This study highlights the effectiveness of lures, however, also indicates that lure effects can vary across genera. Therefore, lures may bias survey results by altering the species composition of bats caught. Future research should focus on a single species or genus, using synthesized calls of conspecifics, to fully understand the effect of lures.

## Graphical abstract

Bat lures have been developed on the premise that broadcasting an acoustic stimulus can increase the number of bat captures in harp traps or mist nets. However, this is still a relatively new, niche method. This is the first time an acoustic bat lure has been systematically tested in a tropical rainforest; assessing the efficacy of two commonly used devices, broadcasting two different acoustic stimuli, in order to increase tropical forest understory bat captures.

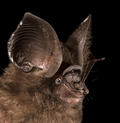

## 1 INTRODUCTION

Bats around the world provide valuable ecosystem services such as pollination (Bumrungsri et al., 2013; Frick et al., 2017), seed dispersal (Kunz et al., 2011; McConkey & Drake, 2006; Mello et al., 2011), and pest control (Baroja et al., 2019; Kemp et al., 2019; Kolkert et al., 2020), while also being bioindicators of environmental degradation and pollution (Jones et al., 2009, 2013; Stahlschmidt & Brühl, 2012). Thus, there is a need for accurate assessments of bat abundance and distribution using standardized survey methods, especially in species‐rich tropical bat communities (Francis, 1990; Nurul‐Ain et al., 2017).

Acoustic lures have been successfully used to increase capture rates in birds (Schaub & Jenni, 1999) and their use in bat surveys has been recommended by some (Hill et al., 2015; Hill & Greenaway, 2005). Several studies from the temperate zone indicate that using bat lures, particularly with social calls, can increase capture rates (Braun de Torrez et al., 2017; Goiti et al., 2008; Hill et al., 2014, 2015; Hill & Greenaway, 2005; Loeb & Britzke, 2010; Quackenbush et al., 2016; Samoray et al., 2018). Methods and results, however, differ significantly, and lure use in tropical forests has not been fully explored.

Early behavioral research on the responses of microchiropteran bats to the playback of ultrasound calls noted that some bats are attracted to the source of the sound (Barclay, 1982; Russ et al., 1998; Wilkinson & Boughman, 1998). Attraction to conspecific acoustic stimuli is widespread in other taxa such as birds (Ndlovu, 2018; Zuberogoitia et al., 2020), amphibians (Gerhardt, 1994), and even fishes (Gordon et al., 2019). Responses of bats to broadcast calls have been explained as mobbing behavior in response to a distress call (Russ et al., 1998), “eavesdropping” on conspecifics echolocation calls to locate resources (Barclay, 1982), in response to social “screeches” acting as contact calls that assist foraging between group members (Wilkinson & Boughman, 1998), for mate choice (Puechmaille et al., 2014), or some combination of the above.

Acoustic bat lures have been developed on the premise that broadcasting acoustic stimuli, generally synthesized bat calls, can attract bats toward traps and therefore increase the probability of capture. This project is the first time an acoustic lure has been systematically tested in a tropical rainforest. It is also the first time that devices from two different manufacturers have been directly compared. Further research is particularly required in the tropics as these are biodiversity hotspots and current lure research has a limited geographic range.

This project aimed to assess the effectiveness of acoustic lures, alongside harp traps and mist nets, for catching insectivorous forest understory bats (narrow‐space foragers; (Schnitzler et al., 2003) in Brunei Darussalam, Borneo. Furthermore, this study compared two devices and two broadcast call types. We tested three hypotheses surrounding lure use: (1) Acoustic lures increase overall bat captures; (2) The Sussex AutoBat lure and the Apodemus BatLure device do not differ in effectiveness; and (3) Using social calls as a lure is more effective than using feeding calls.

## 2 MATERIALS AND METHODS

**FIGURE 1:**
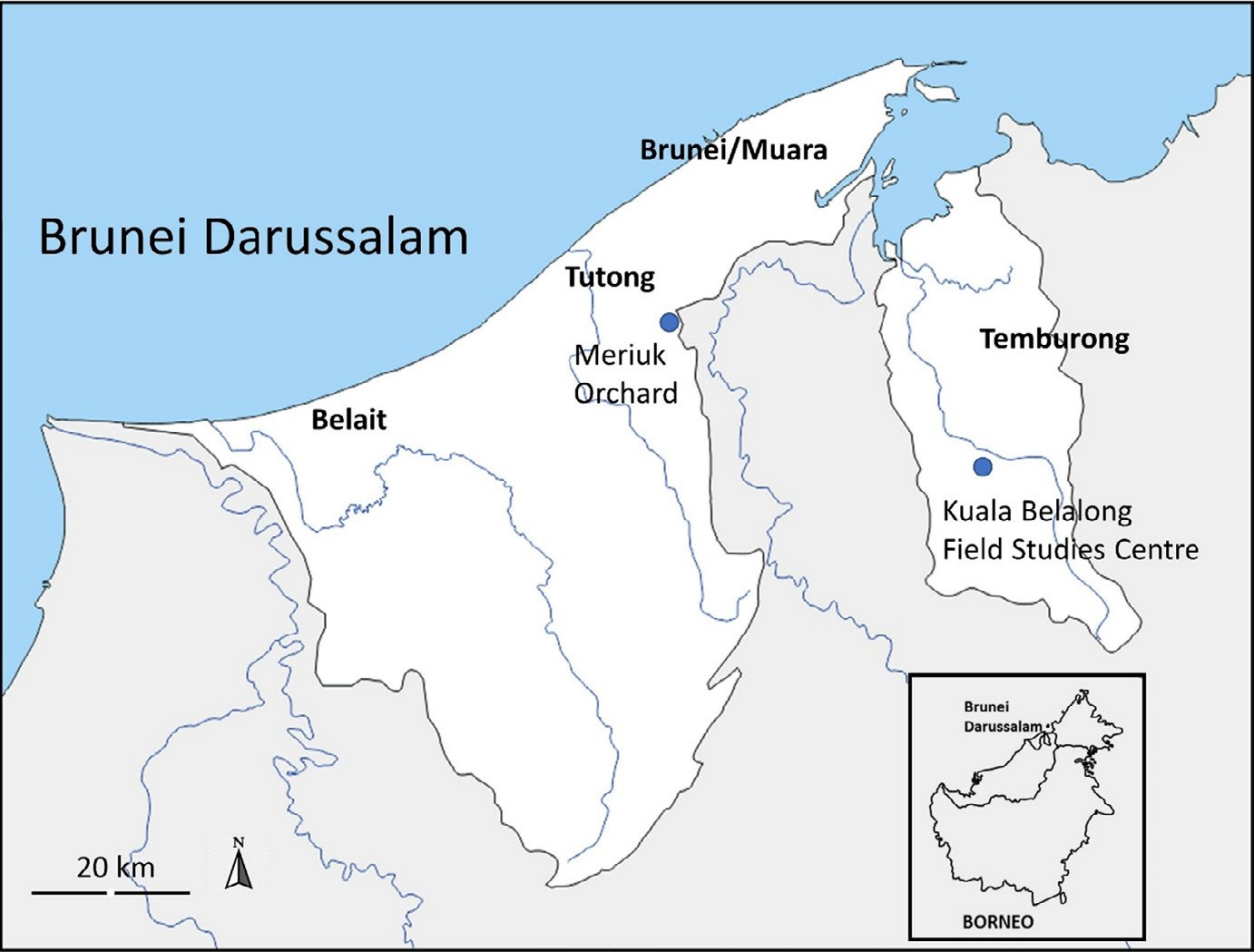
Location of the Kuala Belalong Field Studies Centre and the Meriuk Orchard within Brunei Darussalam. The two sites are 47 km apart. Inset shows the location of Brunei Darussalam on the island of Borneo, Southeast Asia

The efficacy of lure use was tested in Brunei Darussalam, a small country located on the northwest coast of Borneo, over half (54%) of which is still covered by unlogged forest (Bryan et al., 2013). The study was carried out at two sites: the Kuala Belalong Field Studies Centre (KBFSC) in the Ulu Temburong National Park, and a forest‐farm mosaic in Tutong District (Figure 1).

The Ulu Temburong National Park is the largest protected area in Brunei Darussalam, located in the eastern portion of the country within the Temburong District. Protected since 1991, it encompasses 50,000 ha of hilly lowland mixed‐dipterocarp rainforest (Struebig et al., 2012; Sukri et al., 2012). The field center is located at the northern end of the park (N4.551901, E115.155703) and managed by Universiti Brunei Darussalam. The center has one main walking track, a 2.4 km loop that climbs approximately 150 m on the west ridge. This is known as the “Ashton Trail” and has the largest bat inventory recorded in Brunei (Kofron, 2002; Masmin et al., 2016; Struebig et al., 2010, 2012). A total of 36 trapping nights (9 field nights x 4 traps per night) were conducted at KBFSC between January 26 and February 6, 2020.

An additional eight trapping nights (2 field nights × 4 traps per night) were undertaken on February 14 and 18, 2020, at Meriuk Fruit Orchard, located near the village of Mungkom in Kampong Kiudang, Tutong District (N4.7182072, E114.7715742). Meriuk Fruit Orchard is a mosaic of recently converted agricultural land (for fruit trees), interspersed by secondary and primary mixed‐dipterocarp forest. A small cave roost containing several bat species is located within 2 km of the fruit orchard.

At both sites, heavy rain impeded many nights of trapping, deterring bat emergence (Geipel et al., 2019; Kunz & Parsons, 2009). Nights on which no bats were caught were not included in the analyses; acoustic detection devices (AnaBat Walkabout, Titley‐Scientific, Brendale, Australia) also indicated no detectable bat activity during these times.

### 2.1 Trap placement

**FIGURE 2:**
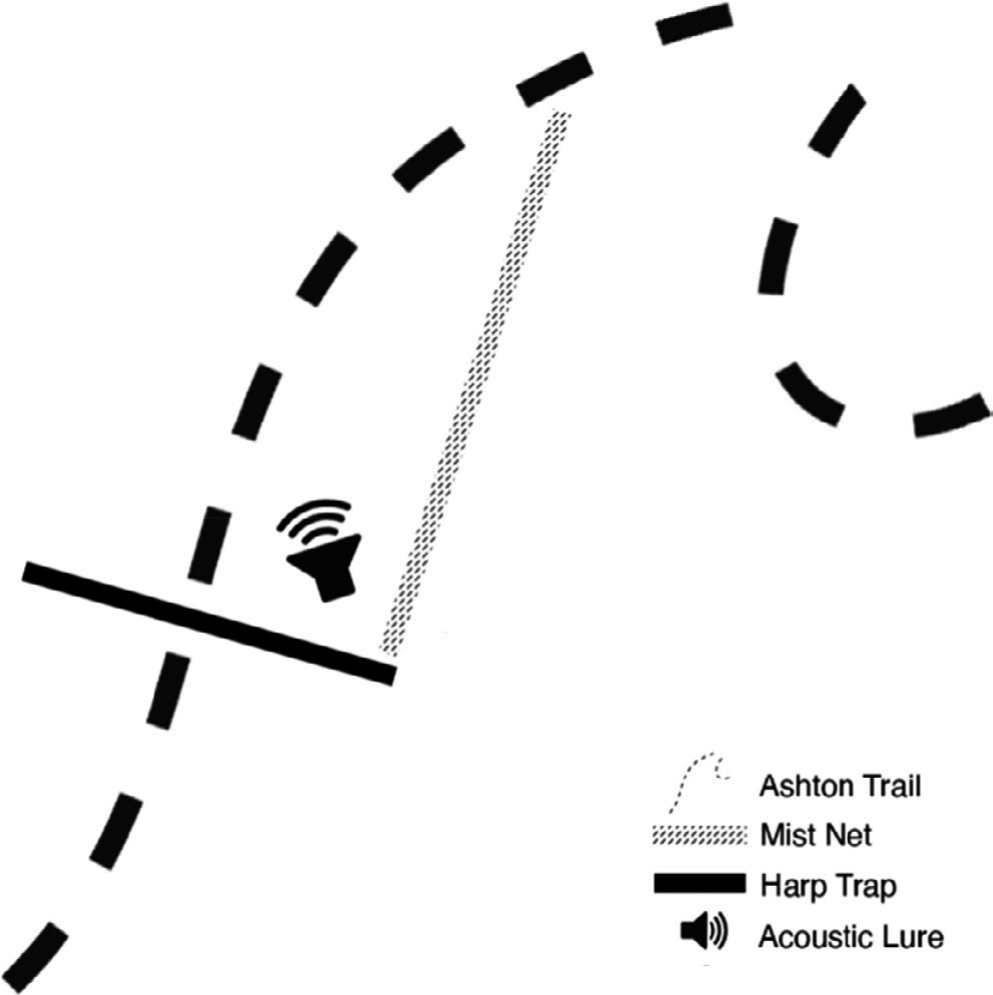
Trap arrangement showing the configuration of harp trap, mist net, and acoustic lure. Two sites, at least 50 m apart, were setup per night using this configuration; a four‐bank harp trap across the existing track or presumed flight path, and a mist net parallel to the track. Each site had an acoustic bat lure mounted on a tripod, either an Apodemus BatLure or a Sussex AutoBat, alternating hourly (control and treatment) throughout the night from 6:00 a.m. to 10:00 p.m. Figure not to scale

Each night two trapping sites, placed at least 50 m apart, were set up along the Ashton Trail (KBFSC) or along the forest‐edge border of the Meriuk orchard. Each site consisted of two traps: a custom‐made four‐bank harp trap placed perpendicular to the established trail (2 cm line spacing, 0.6 mm nylon line, aluminum frame 1.5 × 1 m banks [Malaysia]), and a mist net (6 × 2.5 m, 5 shelved, 16 × 16 mm mesh; Ecotone, Gdynia, Poland [en.ecotone.com.pl]) placed parallel to the track (Figure 2). Harp traps were placed to increase likelihood of captures as bats often use existing tracks to navigate dense forest (Kunz & Parsons, 2009).

In contrast, the mist nets were placed parallel to the track to be less conspicuous against the vegetation and potentially more likely to catch understory bats. Each site was used as both control and treatment to remove any site bias. Morphological measurements (forearm length, weight, and photos) were taken in the field and individuals were identified to species using “Phillipps field guide to the mammals of Borneo and their ecology” (Phillipps & Phillipps, 2018). Bats were identified to genus, but pooled for analysis due to small sample size.

### 2.2 Lure devices

We tested two of the three most commonly used lure devices (Aylen, 2021). The Sussex AutoBat has been tested more thoroughly in the field within the published literature (Goiti et al., 2008; Hill et al., 2014, 2015; Hill & Greenaway, 2005; Lintott et al., 2014) compared to devices from all other manufacturers (Braun de Torrez et al., 2017; Loeb & Britzke, 2010; Quackenbush et al., 2016; Samoray et al., 2018), although a survey of lure users suggests that the Apodemus BatLure and Binary Acoustics AT100 have been used more often (Aylen, 2021).

The Apodemus BatLure uses a single Vifa XT25SC90‐04 omni‐directional speaker (additional speaker available for purchase), whereas the Sussex AutoBat utilizes two SensComp Series 600 Environmental Grade Electrostatic Ultrasonic Sensor's mounted back to back, making the speaker bi‐directional (see Aylen[, 2021] for more information on the available lure devices and specifications). The technical specifications of these devices are similar, despite using different speakers, with both capable of broadcasting between 20 and 100 kHz and optimized for frequencies between 40 and 60 kHz (batmanagement.com; David Hill, personal communication).

### 2.3 Lure setup

An acoustic lure was mounted on a tripod approximately 1–1.5 m off the ground at an approximately 110 degree angle, from 6:00 to 10:00 p.m. each night to coincide with greatest bat activity (Figure 2). The Apodemus BatLure was programmed to match the Sussex AutoBat existing playback settings (not standard from manufacturer), as the Sussex AutoBat playback settings cannot be easily altered. The selected broadcast call played twice followed by 5 s of silence, and this repeated continuously for 15 min.

Every 15 min the broadcast call was manually changed, resulting in 1–2 min of silence. In addition, every 30 min the device was alternated between the Apodemus BatLure and the Sussex AutoBat, and every hour the lures were alternated between treatment and control traps/nets. To ensure each broadcast call and device was used during each time period, the starting device and call type was alternated nightly using two sequences (Table 1).

**TABLE 1:** Setup nightly sequence of lure device, broadcast call, and time past the hour. The sequence alternated between “Sequence 1” and “Sequence 2” each field night to ensure each time block had every combination of device and call

Time	Sequence 1		Sequence 2	
	Device	Call	Device	Call
‐:00	Apodemus	Feeding	AutoBat	Social
‐:15	Apodemus	Social	AutoBat	Feeding
‐:30	AutoBat	Feeding	Apodemus	Feeding
‐:45	Autobat	Social	Apodemus	Social

### 2.4 Broadcast calls

**FIGURE 3:**
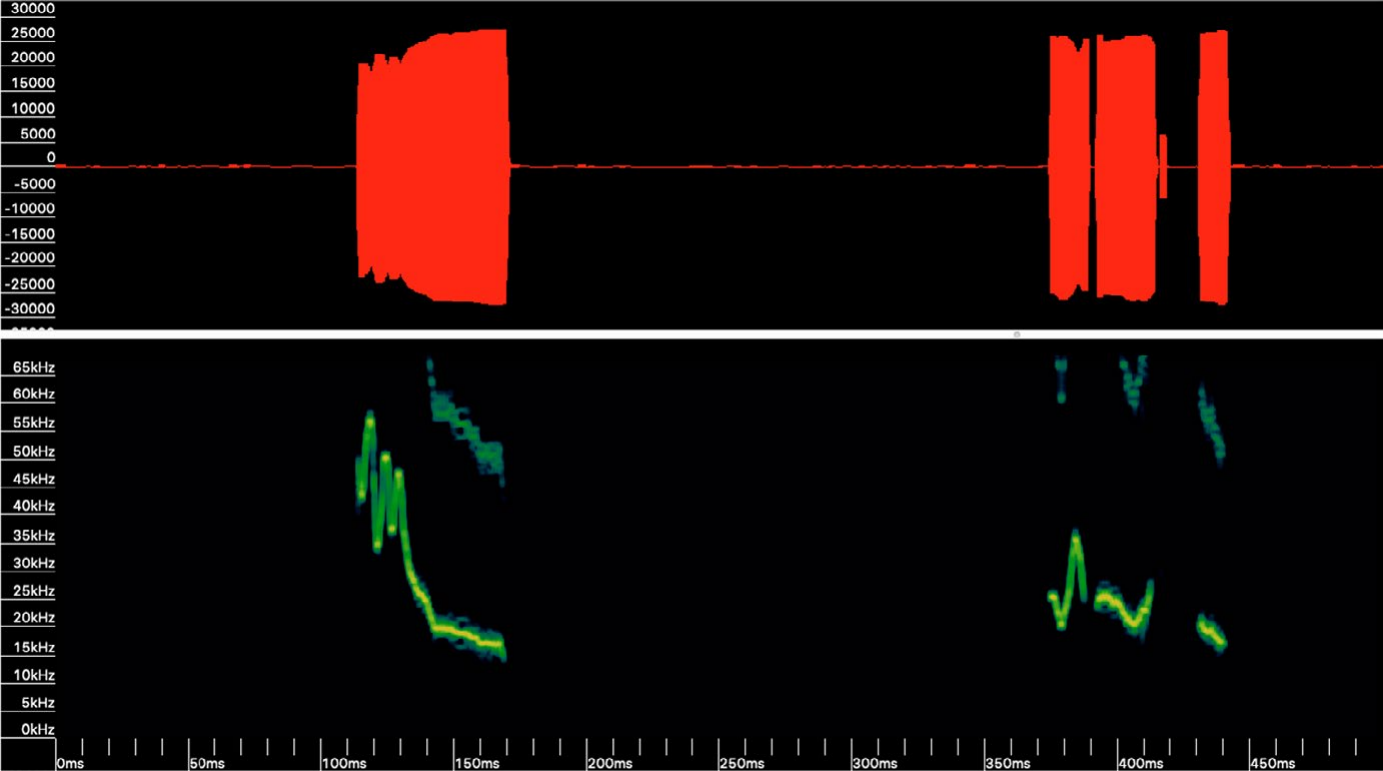
Lure broadcast call 1. Synthesized social call (unknown function) of *Chalinolobus nigrogriseus* with two main parts: an approximately 60 ms long sinusoidal FM call from 60 to 15 kHz, followed by three shorter pulses ranging from 38 to 15 kHz of 65 ms duration, both with a subtle harmonic. FFT 1024. Call synthesized and provided pre‐programmed onto the Sussex AutoBat by Dr. Roger B. Coles

**FIGURE 4:**
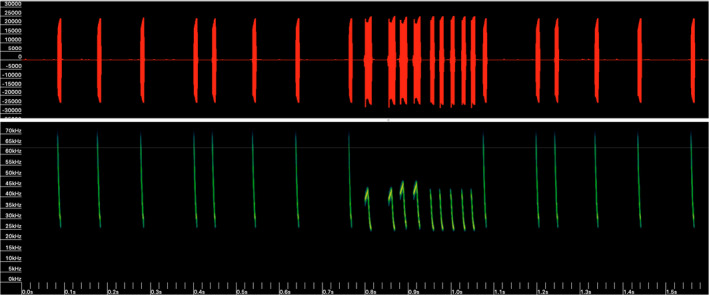
Lure broadcast call 2. Synthesized feeding call of *Myotis macropus* with three parts: an initial 0.77 s long search‐phase composed of several FM sweeps from 70 to 25 kHz, followed by a shorter 0.2 s call of several FM sweeps from 48 to 25 kHz with the initial four pulses having a short up‐sweep, and finishing with a 0.5 s search phase composed of several FM sweeps from 70 to 25 kHz. FFT 1024. Call synthesized and provided pre‐programmed onto the Sussex AutoBat by Dr. Roger B. Coles

Two synthesized Australasian species’ calls were used as broadcast lures on each device. These were pre‐programmed onto the Sussex AutoBat by Dr. Roger B. Coles (Queensland University of Technology). These species would be considered “foreign” as they do not occur in Brunei. One call was based on a social call (unknown function) of *Chalinolobus nigrogriseus* with two main parts: an approximately 60‐ms‐long sinusoidal FM sweep from 60 to 15 kHz, followed by three shorter semi‐sinusoidal components ranging from 38 to 15 kHz, both with a subtle harmonic (Figure 3). The other was based on a feeding call of *Myotis macropus* with three parts. An initial 0.77‐s‐long search‐phase composed of several FM sweeps from 70 to 25 kHz, followed by a 0.26 s of several FM sweeps from 48 to 25 kHz with the initial four pulses having a short up‐sweep, and finishing with a similar 0.5 s search‐phase composed of several FM sweeps from 70 to 25 kHz (Figure 4). Original calls were recorded by Dr. Roger B. Coles in open savannah woodland on Cape York Peninsula, Northern Australia, with an S‐25 UltraSoundAdvice bat detector.

### 2.5 Statistical analyses

To analyses the effect of an acoustic lure on capture rates of bats, a generalized linear mixed model (“GLMMer”; negative binomial distribution due to overdispersion) was run in Program R (R v4.0.3; R Core Team, 2019) with the R package collection “tidyverse” (Wickham et al., 2019) and “lme4” (Bates et al., 2015). Night and trap site were included as random effects. The response variable was number of bats caught per hour as treatment and control nets were changed hourly. The model included total trapping effort, including time periods with no captures, as these are true random zeros (Blasco‐Moreno et al., 2019) within the sampling variability (*n* = 88; 11 field nights × 2 trapping sites × 4 h per night). We then analyzed the number of bats caught only when a lure was broadcasting to assess any effect of the device used or call type broadcast using Chi‐squared tests (*n* = 24).

## 3 RESULTS

In total, 35 individual bats from five genera were captured. Over twice as many bats were caught when either the Sussex AutoBat or Apodemus BatLure was present (24 individuals) rather than with no lure present (11 individuals; *n* = 88; number of bats caught per hour; Table 2); the use of a lure increased capture rates of bats significantly (GLMM, *df* = 1, *p* = .03). Analysis on the number of bats caught only when a lure was broadcasting (*n* = 24) showed no significant difference in captures based on which device was broadcasting ($\chi^{2}$ = 3.24, *df* = 1, *p* = .07). There was also no significant difference in capture rates based on whether the social or feeding call was being broadcast ($\chi^{2}$ = 0.36, *df* = 1, *p* = .5). There were too few captures to statistically analyse any effect of the lure on species or genus, however, 79% of *Kerivoula* sp. (*n* = 14) were caught when a lure was present. In contrast, 71% of *Rhinolophus* sp. (*n* = 7) were caught when there was no lure present. The majority of *Hipposideros* sp. were caught when a lure was present (67%; *n* = 6), and this was equally distributed between the two broadcast calls. Two *Phoniscus* sp. and four *Murina* sp. were caught, all when a lure was broadcasting. Only 17% of bats caught (*n* = 35) were captured in mist nets, the remaining 83% being captured in harp traps (Table 2).

**TABLE 2:** Number of individual bats captured over 44 trapping nights, sorted by genus. Number of bats captured without a lure, numbers caught using a lure (Apodemus BatLure vs. Sussex AutoBat), numbers caught using different types of broadcast calls (feeding vs. social), and numbers caught per trap type (harp trap or mist net) are shown. Two bats escaped traps before they could be identified. Bats were pooled for analysis due to a small sample size

Genus	# of Bats	No Lure	Lure Device		Broadcast Call		Trap Type	
			Apodemus	AutoBat	Feeding	Social	Harp	Mist
*Kerivoula*	14	3	1	10	5	6	13	1
*Rhinolophus*	7	5	0	2	2	0	4	3
*Hipposideros*	6	2	3	1	2	2	6	0
*Murina*	4	0	2	2	2	2	3	1
*Phoniscus*	2	0	1	1	1	1	2	0
Escaped	2	1	0	1	1	0	1	1
Total	35	11	7	17	13	11	29	6

## 4 DISCUSSION

Lure use had a significant positive effect on capture rates, with more bats captured when a lure was broadcasting (Table 2). Approximately two‐thirds of the bats captured (24 of 35) were captured while an acoustic lure was in use. This adds to the growing pool of research highlighting the effectiveness of acoustic lure devices in surveying forest understory bats (Goiti et al., 2008; Hill et al., 2015; Hill & Greenaway, 2005; Lintott et al., 2014). The type of device made no difference to capture rates. Likewise, the type of call broadcast and feeding call versus social call also had no effect on capture rates, with a near even distribution between the two call types (Table 2). This result suggests that call type may be less important than often assumed. However, since this study utilized calls modeled on two different “foreign” Australasian species, it is likely the bats are responding to the presence of a novel sound rather than out of interest in communicating with conspecifics whose calls generally differ substantially (Khan et al., 2021).

One major difference between the lure devices is that the Sussex AutoBat has a bidirectional speaker, which was likely slightly inhibited on one side through being placed at an approximately 110 degree angle (one speaker pointed more toward the ground). As well as the difference in speaker directionality, the Apodemus BatLure can be attached to a tripod for ease of deployment, and broadcast calls are easily changeable via an SD card that can be loaded with chosen audio (.wav) files. Overall, the technical specifications are similar as both are able to broadcast between 20 and 100 kHz and optimized for frequencies between 40 and 60 kHz (batmanagement.com; David Hill, personal communication), although the designs differ substantially (Aylen, 2021). Our results show that these devices are equally as effective with our chosen broadcast calls; both of which fell within/below the optimized frequency range. It is, however, possible that this may not be the case when broadcasting calls >60 kHz, as it has been suggested that some devices are more effective at higher frequencies (batmanagement.com; Aylen, 2021).

A result of our study, and of particular interest, is the lack of difference in effectiveness between the two broadcast call types: feeding calls versus social calls. Similarly, no differences in captures rate were seen in playback experiments on vespertilionid bats in the USA (Quackenbush et al., 2016; Samoray et al., 2018), while differences were observed in Scotland, although no details on the specific calls used were given (Lintott et al., 2014). Calls were significantly different in studies showing no effect, and in theory, each call represents the presence of a food source, food competition, or an unknown social stimulus. These results support the theory that the primary attraction of lures is merely the presence of a novel sound, rather than a form of communication or eavesdropping, a topic that is still debated.

Our study utilized foreign Australasian calls for our lure devices. Some studies have suggested that using a local conspecific call is most appropriate (Hill et al., 2015; Schöner et al., 2010), whereas others suggest a foreign and/or novel sound is more appropriate (Hill & Greenaway, 2005; Lintott et al., 2014; Quackenbush et al., 2016). However, even within a single region, the calls of some species can show considerable geographic variation (Sun et al., 2020) and many other factors are likely to be relevant, including the background bat activity when a lure is deployed (Lewanzik et al., 2019). In some cases, using conspecific calls has been unsuccessful in attracting the target species, having instead attracted other bats (Lintott et al., 2014). Future studies would likely benefit from focusing on one species to fully understand the effect of the lure and the most appropriate broadcast call(s), if any.

In our study, the lures were mostly ineffective at attracting horseshoe bats (*Rhinolophu*s sp.), possibly even deterring them. It has been suggested that lures are less effective for bats of the genus *Rhinolophus* (Jon Flanders, personal communication), although there is no specific evidence as of yet. The two most common *Rhinolophus* sp. at KBFSC are *Rhinolophus trifoliatus* and *Rhinolophus sedulous*. Of the total bats caught in this genus, 71% of them were caught when no lure was present (Table 2). Our results suggest that the lure possibly deterred them from trap sites, potentially acting as signs of intraspecific competition for foraging areas, as has been suggested with the North American vespertilionid bat *Corynorhinus rafinesquii* (Loeb & Britzke, 2010).

Bats in the genus *Murina* have been observed to be particularly attracted to lures in Japan (Hill et al., 2014). Hill et al. (2014) suggest this is due to these species being narrow‐space foraging bats, making them otherwise difficult to catch with standard methods (harp traps or mist nets positioned across flyways). A high proportion of *Kerivoula* (79%) were caught in traps with a lure (Table 2), and this was the most abundant genus caught overall. Since both *Murina* and *Kerivoula* are “narrow space passive gleaning foragers” (Denzinger & Schnitzler, 2013) with particularly high‐frequency calls compared to other species in the bat assemblage, it may have been that the acoustic stimulus indicated to these species, in contrast to the horseshoe bats, a profitable foraging site or similar source of interest. Gaining such information through eavesdropping is a known behavior in other bats (Fenton, 2003; Gillam, 2007; Ubernickel et al., 2013).

More recently, lures have also been used successfully to catch rare “open‐space aerial foragers” (Braun de Torrez et al., 2017). Given the significantly high capture rates observed elsewhere, and our results, it would be interesting to focus on this foraging guild in particular for future research as such bats are often underrepresented in faunal surveys. Furthermore, despite only two *Phoniscus atrox* being caught in this study, both were caught in traps with a lure and is a promising result that could potentially lead to increased captures of this near threatened species (Jayaraj, 2020).

Use of a lure to capture bats in Europe, Australia, and Japan has also been successful with 12–24.5x more captures in lure traps (Hill et al., 2014, 2015; Hill & Greenaway, 2005; Lintott et al., 2014). The above studies, however, did not place traps across flyways, as is often standard practice for catching forest bats (Kunz & Parsons, 2009), instead avoiding them. Avoiding flyways limits incidental catches, those likely to occur regardless of lure presence (unless the lure acts as a deterrent), however, potentially overinflates the effect of the lure compared to standard methods, that is, utilization of flyways. Appropriate trap placement is likely to impact bat captures more than incorporating a lure, with less general setup and financial cost required. Lower efficacy of lures occurs when standard trap placement is used (Quackenbush et al., 2016; Samoray et al., 2018). While we placed mist nets against the forest edge, the lower number of bats captured in mist nets is more likely due to bats being better able to detect and avoid mist nets (Kingston et al., 2003).

A further advantage of our study is the use of synthesized calls which allows for simple standardization of methods, and increases output clarity since no background noise is included in the playback. In general, synthesized calls are recommended for use with acoustic lures (Hill et al., 2015), and have been primarily used in many published lure studies (Goiti et al., 2008; Hill et al., 2014; Lintott et al., 2014; Quackenbush et al., 2016). We also provide full details of call parameters and the spectrograms to facilitate full use or modification in future studies. This is an important piece of information to make available for reproducibility, and to increase depth of understanding, but is not always provided in other publications.

## 5 CONCLUSIONS

We tested three hypotheses in this study: (1) using an acoustic lure would increase overall bat captures; (2) the Sussex AutoBat lure device and the Apodemus BatLure devices do not differ in effectiveness; and (3) using social calls as a lure is more effective than using feeding calls. Overall, lures significantly increased capture rates, while the specific device and broadcast calls used did not measurably differ. The two broadcast call types had an even distribution of captures, supporting the notion that a novel sound may be more attractive than the specific call type. *Murina* (100%), *Phoniscus* (100%), *Hipposideros* (67%), and *Kerivoula* (79%) were caught more often in traps with a lure, than without, indicating greater attraction to lures in these bats. In contrast, 71% of *Rhinolophus* species were caught in traps when no lure was present, suggesting a deterrent effect. Future research should focus on a single species or genus to fully understand the effect of the lure and broadcast stimuli, as its effectiveness appears to vary between bat genera. Manufacturers would also benefit from providing/supporting more published field tests on the appropriate methods and calls to use with their lure devices. Lastly, our study provides evidence that the use of a lure may alter the species composition of bats caught, increasing captures of some genera, while deterring others, and thus, may bias survey results.

## CONFLICT OF INTEREST

The authors declare no conflict of interest.

## AUTHOR CONTRIBUTION

**Oliver Aylen:** Conceptualization (lead); Data curation (lead); Formal analysis (lead); Methodology (lead); Project administration (lead); Resources (equal); Software (lead); Writing – original draft (equal); Writing – review & editing (equal). **Phil J. Bishop:** Conceptualization (equal); Funding acquisition (lead); Investigation (equal); Methodology (equal); Project administration (equal); Resources (equal); Supervision (lead). **Rodzay bin Haji Abd Wahab:** Project administration (equal); Resources (equal); Writing – review & editing (equal). **T. Ulmar Grafe:** Conceptualization (equal); Data curation (equal); Formal analysis (equal); Methodology (equal); Project administration (equal); Resources (equal); Supervision (equal); Writing – original draft (equal); Writing – review & editing (equal).

## Data Availability

The data that support the findings of this study are openly available at Dryad archiving: https://doi.org/10.5061/dryad.63xsj3v4c.
